# Supplementary figures and images for: NINscope, a versatile miniscope for multi-region circuit investigations
Source: eLife. 2020 Jan 14;9:e49987. doi: 10.7554/eLife.49987 (PMC6989121; doi:10.7554/eLife.49987)

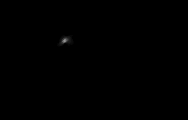

Supplement: Figure 4—source data 1. [file elife-49987-fig4-data1.zip › Figure4-source data/Figure4_panel_F_components.tif]
